# Severe Dilated Cardiomyopathy with PLACK Syndrome Caused by a Novel Truncating Variant in the CAST Gene

**DOI:** 10.3390/genes16111292

**Published:** 2025-10-30

**Authors:** Maarab Alkorashy, Hamzah Naji, Nadiah ALRuwaili, Dimpna Albert, Saud Takroni, Shamayel Mohammed, Hadeel Binomar, Aisha ALqahtani, Zuhair Al-Hassnan

**Affiliations:** 1 Department of Translational Genomics, Genomic Medicine Center of Excellence, King Faisal Specialist Hospital & Research Centre, Takhassusi Street, P.O. BOX 3354, Riyadh 11211, Saudi Arabiahanaji@kfshrc.edu.sa (H.N.); aalqahtani51@kfshrc.edu.sa (A.A.); 2 Heart Center of Excellence, King Faisal Specialist Hospital & Research Centre, Riyadh 11211, Saudi Arabia; 3 Department of Pathology and Laboratory Medicine, King Faisal Specialist Hospital & Research Centre, Takhassusi Street, P.O. BOX 3354, Riyadh 11211, Saudi Arabia

**Keywords:** *CAST* gene, dilated cardiomyopathy, PLACK syndrome, heart transplantation

## Abstract

**Background:** PLACK syndrome is an ultra-rare autosomal recessive disorder caused by biallelic loss-of-function variants in *CAST*, which encodes calpastatin, an endogenous inhibitor of calpains. The syndrome is classically defined by peeling skin, leukonychia, acral punctate keratoses, cheilitis, and knuckle pads. Although the phenotype has been largely restricted to dermatological manifestations, emerging reports suggest dilated cardiomyopathy (DCM) as a systemic complication. **Methods:** We investigated five affected children from three sibships of an extended consanguineous family. Clinical evaluation and genome sequencing (GS) followed by segregation analysis of the targeted mutation test (TMT) were performed. Histopathological examination of an explanted heart was conducted in one child who underwent heart transplantation. **Results:** All affected children exhibited typical dermatological features of PLACK syndrome. Four developed severe DCM, two of whom required orthotopic heart transplantation. GS, performed in three affected children, identified a novel homozygous frameshift variant in *CAST* (NM_001750.7:c.1177dup, p.Arg393Profs*4), which segregated with the disease within the family. No additional plausible variants in known cardiomyopathy-associated genes were detected. Histopathological examination of the explanted heart demonstrated hypertrophied cardiomyocytes with nuclear enlargement, hyperchromasia, and fibrosis. **Conclusions:** Our findings expand the phenotypic spectrum of PLACK syndrome to include severe DCM and suggest *CAST* deficiency as a novel cause of recessively inherited cardiomyopathy. The favorable short-term outcome following transplantation highlights a potential therapeutic option. Given the possibility of age-dependent penetrance, lifelong cardiac surveillance is for the affected individuals suggested. To emphasize cardiomyopathy as a critical and underrecognized component of the syndrome, we propose the consideration of modifying the acronym to PLACK-C.

## 1. Introduction

PLACK syndrome, caused by biallelic loss-of-function (LOF) variants in the *CAST* gene, is an ultra-rare autosomal recessive disorder characterized by the following defining dermatologic features: peeling skin, leukonychia, acral punctate keratoses, cheilitis, and knuckle pads (PLACK) [1]. Calpastatin, the protein encoded by *CAST*, is an endogenous inhibitor of calpains, which are non-lysosomal calcium-dependent cysteine proteinases found in all mammalian cells [2,3].

To date, 21 pediatric and adult cases (Supplemental Table S1) affected with PLACK syndrome have been reported in the literature with a phenotype that is mostly confined to the typical dermatological manifestations [4,5]. Recently, dilated cardiomyopathy (DCM) has emerged as a potential systemic manifestation of PLACK syndrome [6,7,8], raising concerns about the impact of *CAST* deficiency on myocardial function.

In this study, we describe five children in three sibships of an extended consanguineous family with PLACK syndrome caused by a novel homozygous frameshift variant in CAST. Four of the affected children developed severe DCM that necessitated heart transplantation in two individuals, offering a rare opportunity for histopathological examination of the explanted myocardium. Our findings suggest that biallelic LOF variants in *CAST* predispose to severe DCM.

## 2. Methodology

### 2.1. Patients

Clinical evaluations were performed on the index cases, their parents, and unaffected siblings. Blood samples were collected from the index cases and their first-degree relatives. Patients were enrolled through the Cardiovascular Genomics Program at King Faisal Specialist Hospital & Research Centre (KFSH&RC). Participation in the study was contingent upon obtaining written informed consent.

### 2.2. Genetic Testing

Genome sequencing (GS), conducted in three of the affected cases, was performed using the Illumina platform, generating fragmented genomic DNA sequences with an average coverage depth of approximately 30×. Low-quality reads were filtered out, and the remaining variants were annotated. The analysis also included the assessment of copy number variations and non-coding variants. The allele frequency of the detected variants was reviewed against the gnomAD database, as well as the Genomic Medicine Centre of Excellence database, which contains the data of more than 18,300 exome and genome sequencings. Then, a targeted mutation test (TMT) was conducted for the detected variants on all available family members, both affected and unaffected. The sample was analyzed using a clinically validated sequencing protocol, and a PCR-based method was used to amplify the specific region of the *CAST* gene containing the targeted variant. The resulting PCR product was subjected to direct Sanger sequencing in both forward and reverse directions, utilizing automated fluorescent dideoxy sequencing technology (further details of the methods of genetic testing can be found in the Supplementary Materials).

### 2.3. Histopathological Study

The histopathology of the explanted heart of the index case (V.12) was examined. Formalin-fixed, paraffin-embedded (FFPE) tissue blocks were cut into 4–5 µm thick sections using a rotary microtome and mounted on glass slides. The sections were deparaffinized in xylene and rehydrated through graded alcohols to distilled water. Standard hematoxylin and eosin staining was performed: sections were stained in hematoxylin, followed by counterstaining with eosin. After dehydration through graded alcohols and clearing in xylene, the slides were mounted with coverslips. Stained sections were examined under a light microscope by a pathologist.

## 3. Results

### 3.1. Clinical Presentation

**Figure 1:**
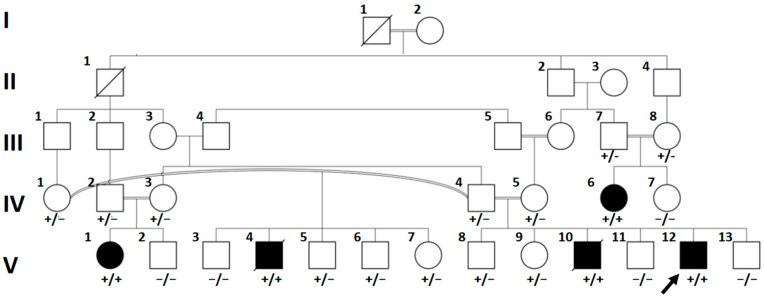
Pedigree of the family with PLACK syndrome. Affected individuals are indicated by filled symbols. The index patient (V.12) is marked with an arrow. Genotypes are shown below each tested individual: wild-type (−/−), heterozygous carrier (+/−), and homozygous affected (+/+). Multiple affected siblings (V.1, V.4, IV.6, V.12) demonstrate the segregation of the pathogenic variant, while obligate carriers are unaffected. NA: not available.

**Figure 2:**
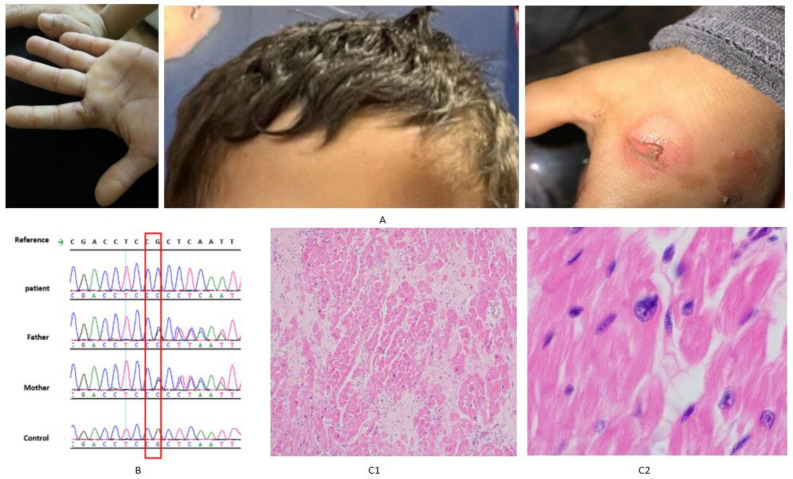
(**A**) Photographs of the index case (V.12) demonstrating hallmark clinical manifestations of PLACK syndrome, taken with consent. (**B**) Sanger sequencing chromatograms showing homozygous *CAST* variant in the index (V12), heterozygous variant in parents (IV4 and IV5), and wild-type sequence in a normal control. (**C**) Histopathology of the explanted heart of the index case (V.12) (**C1**) Section from the left ventricle showing hypertrophied myocytes on a fibrous background. (**C2**) Generalized enlargement of hyperchromatic and bizarrely shaped nuclei.

A 7-year-old male patient (individual V.12, Figure 1) was referred to the Cardiovascular Genomics Program at King Faisal Specialist Hospital and Research Centre (KFSH&RC) with a clinical diagnosis of DCM and hyperkeratosis. Dermatological manifestations had been apparent since the first six months of life and included focal hyperkeratosis of the palms and soles, cutaneous warts, punctate palmoplantar keratoderma, and silver hair (Figure 2A). He was diagnosed with DCM at the age of 6 years. His cardiac condition progressed with heart failure that necessitated orthotopic heart transplantation at the age of seven years. He is currently five months post-transplantation and his condition has been well.

Family history reveals that the parents are consanguineous and clinically unaffected. The father is married to two women, both of whom are his first cousins. A proband’s half-brother (individual V.4, Figure 1) died at eight years of age from DCM with end-stage heart failure while awaiting heart transplantation, and another brother (individual V.10, Figure 1) died at nine years of age from a similar cardiac condition. Both brothers had the characteristic skin features of PLACK syndrome. Two additional relatives (individuals IV.6 and V.1, Figure 1) exhibited dermatological manifestations in early infancy. Individual IV.6 was diagnosed with DCM at the age of 10 years and underwent heart transplantation. She is alive and clinically stable one-year post-transplantation. Individual V.1, currently four years old, has been undergoing regular cardiac surveillance and has not shown evidence of cardiac involvement yet.

### 3.2. Genetic Result

GS on individuals V.1, V.12, and IV.6 detected a novel homozygous frameshifting variant (NM_001750.7:c.1177dup, p.Arg393Profs*4) in exon 14 in the *CAST* gene. No other variants in the genes known to cause cardiomyopathy were found. Segregation analysis revealed that the parents of the affected children were heterozygous. Unaffected siblings (*n* = 8) were either heterozygous or homozygous for the wild-type allele (Figure 1). Individual V.4, who is affected, was found to be homozygous for the mentioned variant via TMT. Individual V.10, who is affected, unfortunately died before testing.

### 3.3. Histopathological Study

An examination of the explanted heart from the index case (V.12) demonstrated marked myocardial structural abnormalities. Sections from the left ventricle showed hypertrophied myocytes within a fibrous background (Figure 2(C1)). In addition, nuclei were diffusely enlarged, hyperchromatic, and displayed irregular, bizarre shapes (Figure 2(C1)).

## 4. Discussion

In this study, we report five children in three sibships from an extended consanguineous family who manifested the typical dermatological phenotype of PLACK syndrome. Four of the affected children developed severe DCM, necessitating heart transplantation in two of them. The diagnosis of PLACK syndrome was confirmed by detecting a novel homozygous frameshifting variant in *CAST* in the affected children. The parents in the three sibships were heterozygous, and the unaffected children were either homozygous for the wild-type allele or heterozygous. Of note, none of the affected children in the three sibships developed DCM without the dermatological findings of PLACK syndrome. In addition, no other variant in DCM-causative genes was detected in any of the three GS performed in the affected children. These findings suggest that mutated *CAST* is a strong candidate of a recessively inherited form of DCM in association with PLACK syndrome.

In the literature, there have been 21 cases, mostly children, reported to have PLACK syndrome with a phenotype confined to the typical dermatological manifestations, in the majority of patients [4,5]. However, in addition to our cases, there have been three reports of DCM in cases with PLACK syndrome supporting an association of mutated *CAST* with recessive cardiomyopathy (Supplemental Table S1). The first report was of a 4-year-old female who presented with the classic dermatological features of PLACK syndrome and subsequently died from DCM [6]. She was homozygous for a nonsense variant in *CAST* (c.1882C>T; p.Gln628*). Notably, two of her brothers had similar dermatological features and died suddenly at the ages of 3 and 4 years, respectively, while an 8-year-old sister carrying the same homozygous variant displayed the skin phenotype but had a normal echocardiogram. The second case was a 5-year-old child who exhibited the typical dermatological manifestations without evidence of cardiac involvement [7]. He was homozygous for a splice-site variant in *CAST* (case # 6, Supplemental Table S1). Remarkably, seven of his family members who were affected by PLACK syndrome had sudden cardiac death between the ages of 8 and 14 years. The third case was a 15-year-old male with both DCM and the dermatological phenotype. Genetic analysis revealed compound heterozygosity for a nonsense variant in one allele and a 326,700 bp deletion (Chr5:96037538_96364237) on the other allele (case # 7, Supplemental Table S1), encompassing *CAST* as well as neighboring genes ERAP2 and LNPEP. No OMIM phenotype has been associated with either ERAP2 nor LNPEP [8].

There has been a growing body of evidence linking calpains to the pathogenesis of myocardial remodeling and heart failure, particularly under conditions of cellular stress or injury [9,10]. Together, calpastatin and calpains form an intracellular, non-lysosomal proteolytic system that is widely expressed in human tissues [1]. In animal models, studies have supported a biologically plausible link between calpain–calpastatin system dysregulation and cardiomyopathy. The transgenic overexpression of mitochondria-targeted calpain-1 (CAPN1) in mice (Tg-mtCapn1/tTA^high) led to severe DCM, mitochondrial dysfunction, and early death [10]. Calpain-overexpressing mouse models have demonstrated cardiac enlargement and fibrosis, notably, in Tg-Capn2/tTA mice that develop DCM with chamber enlargement, myocardial fibrosis, and functional decline by 8 months of age [11].

Previously reported variants in *CAST* associated with PLACK syndrome have been truncating in nature (frameshift, splice-site, and nonsense mutations). Reviewing the variants in PLACK syndrome cases that have been reported with DCM reveals that no distinct variant hotspots for cardiomyopathy can been identified. Variants linked to cardiac manifestations affected similar protein domains as those observed in patients without cardiac involvement. This observation suggests that there is lack of genotype–phenotype correlation with respect to cardiac involvement.

In our family, two of the affected children (individuals V.12 and IV.6, Figure 1) who had severe DCM underwent heart transplantation at the age of seven and ten years, respectively, while two others succumbed to their cardiac disease. In the proband’s explanted heart, the histopathological findings were nonspecific. There were hypertrophied cardiomyocytes on a fibrous background with generalized enlargement of hyperchromatic and bizarrely shaped nuclei. To our knowledge, there have been no published histopathology reports of heart tissues in cases with PLACK syndrome. Likewise, histopathological studies showed no specific changes in mice models of calpastatin transgenic hearts, but electron microscopy revealed an extensive sarcomeric disruption, the formation of amorphous protein aggregates, frequent myelin bodies, and abundant autophagic vacuoles [12]. In our family, both children who were transplanted are alive and have been in a stable clinical condition for five months and one year, respectively, after the transplantation. To our knowledge, this is the first report of the outcome of a heart transplantation in DCM associated with PLACK syndrome.

Of note, all of the reported cases of PLACK syndrome with DCM, in addition to our cases, had childhood-onset cardiac disease. Interestingly, the oldest reported cases with PLACK syndrome, who were 54 years and 58 years old, had no cardiac phenotype [1]. In our family, the 4-year-old cousin of the proband (individual V.1, Figure 1) presented with isolated dermatological manifestations and has not developed cardiac involvement as of the most recent evaluation. This indicates that she might be in the presymptomatic stage and hence the cardiac involvement may have an age-dependent penetrance. Similarly, the 4-year-old case with PLACK syndrome and DCM reported by Durmaz et al. [6] had an 8-year-old sister who displayed the skin phenotype with normal echocardiogram. Given these observations of reduced and possibly age-dependent penetrance of DCM in PLACK syndrome and the lack of genotype–phenotype correlation, it would be prudent to advise for a life-long surveillance with echocardiography for patients who have not shown cardiac involvement.

The rarity of PLACK syndrome, which has restricted the number of affected individuals available for detailed analysis, is one of the main limitations of our study. Additionally, while a predicted truncating variant in *CAST* was identified in our cases, functional validation was not performed to verify a causal relationship. Furthermore, additional histopathological evaluation, such as electron microscopy, was not feasible, as the explanted cardiac tissue was formalin-fixed and paraffin-embedded, limiting the ability to assess ultrastructural details.

## 5. Conclusions

Our report suggests that *CAST*-related DCM has a favorable outcome for heart transplantation. Yet, a longer follow-up is needed to better understand the long-term outcomes. Our findings, alongside the available literature, highlight that cardiac manifestation may represent a more common and severe aspect of PLACK syndrome that might be overlooked. To raise awareness of this understudied aspect of PLACK syndrome and underscore the importance of periodic cardiac evaluation, we propose that the acronym PLACK-C might be considered if further cases consistently demonstrate cardiomyopathy. Reporting more cases with careful analysis of the genotype and phenotype would shed more light on our understanding of this ultra-rare syndrome.

## Data Availability

The original contributions presented in this study are included in the article/Supplementary Materials. Further inquiries can be directed to the corresponding author.

## Supplementary Materials

The following supporting information can be downloaded at: https://www.mdpi.com/article/10.3390/genes16111292/s1, Table S1: The demographic and molecular findings of previously reported cases of PLACK syndrome alongside our cohort.

## Abbreviations

PLACK	Peeling skin, leukonychia, acral punctate keratoses, cheilitis, and knuckle pads
DCM	Dilated cardiomyopathy
GS	Genome sequencing
TMT	Targeted mutation test
FFPE	Formalin-fixed, paraffin-embedded
H&E	Hematoxylin and eosin

## References

[1] Lin Z., Zhao J., Nitoiu D., Scott C.A., Plagnol V., Smith F.J., Wilson N.J., Cole C., Schwartz M.E., McLean W.I. (2015). Loss-of-function mutations in CAST cause peeling skin, leukonychia, acral punctate keratoses, cheilitis, and knuckle pads. Am. J. Hum. Genet..

[2] Hanna R.A., Campbell R.L., Davies P.L. (2008). Calcium-bound structure of calpain and its mechanism of inhibition by calpastatin. Nature.

[3] Melloni E., Salamino F., Sparatore B. (1992). The calpain-calpastatin system in mammalian cells: Properties and possible functions. Biochimie.

[4] Haxho F., Haber R.M., Mohamad J., Sarig O., Sprecher E., Perrier R., Boctor D., Ramien M. (2025). Peeling Skin, Leukonychia, Acral Punctate Keratoses, Cheilitis and Knuckle Pads (PLACK) Syndrome: An Updated Review of Cases and Identification of a Recurrent CAST Variant in Two Patients. Pediatr. Dermatol..

[5] Srinivas S.M., Basavapura S. (2025). PLACK Syndrome in Two Unrelated Indian Children Caused by Novel Pathogenic Variants in the CAST Gene. Pediatr. Dermatol..

[6] Durmaz C.D., Tekmenuray-Unal A. (2023). Novel nonsense CAST mutation in two siblings with PLACK syndrome. Int. J. Dermatol..

[7] Mamivand A., Zekri A., Maghrouni A., Bayat S., Mirzaei E., Javadi Golroodbari F., Mousavi S.M., Behrangi E., Tabrizi M. (2023). A patient with PLACK syndrome with a novel splicing mutation in CAST: The evidence for a loss-of-function mechanism through mis-splicing. Clin. Exp. Dermatol..

[8] Nguyen C., Hughes C., Little S., Carruth A., Nolan D., Ruth J. (2024). CASTing the net wider: A case report of PLACK syndrome associated with dilated cardiomyopathy. Pediatr. Dermatol..

[9] Letavernier E., Zafrani L., Perez J., Letavernier B., Haymann J.P., Baud L. (2012). The role of calpains in myocardial remodelling and heart failure. Cardiovasc. Res..

[10] Cao T., Fan S., Zheng D., Wang G., Yu Y., Chen R., Song L.-S., Fan G.-C., Zhang Z., Peng T. (2019). Increased calpain-1 in mitochondria induces dilated heart failure in mice: Role of mitochondrial superoxide anion. Basic Res. Cardiol..

[11] Ji X.Y., Zheng D., Ni R., Wang J.X., Shao J.Q., Vue Z., Hinton A., Song L.-S., Fan G.-C., Chakrabarti S. (2022). Sustained over-expression of calpain-2 induces age-dependent dilated cardiomyopathy in mice through aberrant autophagy. Acta Pharmacol. Sin..

[12] Galvez A.S., Diwan A., Odley A.M., Hahn H.S., Osinska H., Melendez J.G., Robbins J., Lynch R.A., Marreez Y., Dorn G.W. (2007). Cardiomyocyte degeneration with calpain deficiency reveals a critical role in protein homeostasis. Circ. Res..

