# Supplementary material for: Severe Dilated Cardiomyopathy with PLACK Syndrome Caused by a Novel Truncating Variant in the CAST Gene
Source: Genes (Basel). 2025 Oct 30;16(11):1292. doi: 10.3390/genes16111292 (PMC12652073; doi:10.3390/genes16111292)
Supplement: Supplementary file 1 [file genes-16-01292-s001.zip › genes-3956614-supplementary.pdf]

Supplementary Materials

Table S1 Legend: The demographic and molecular findings of previously reported cases of PLACK syndrome alongside our cohort.

#	Age	Sex	PLACK syndrome dermatlogic features	DCM (Yes/No)	Age of onset of DCM (years)	Heart Transplant	*CAST* variant	References
1	7	Male	Yes	Yes	4	Yes	c.1177dup (p.Arg393Profs*4)	This study
2	8	Male	Yes	Yes	6	No	c.1177dup (p.Arg393Profs*4)	This study
3	9	Male	Yes	Yes	8	No	Not tested	This study
4	11	Femal e	Yes	Yes	9	Yes	c.1177dup (p.Arg393Profs*4)	This study
5	4	Femal e	Yes	No	NA	No	c.1177dup (p.Arg393Profs*4)	This study
6	5	Male	Yes	*	8-14 years	No	c.1209+2T>G	PMID:37221445
7	15	Male	Yes	Yes	15	No	c.331C>T hemizygous mutation in one *CAST* allele and deletion of second allele encompassing the entire gene and two neighboring genes, *ERAP2* and *LNPEP* ([GCHr37] Chr5: 96037538_96364237),(p.Gln111Ter)	PMID:38994911

8	4	Female	Yes	Yes	3 years (History of 2 siblings with PLACK, suddenly died at age of 3-4 years) not tested	No	c.1882C>T (p.Gln628Ter)	
9	8	Female	Yes	No	NA	No	c.1882C>T (p.Gln628Ter)	PMID:37387535
10	54	Male	Yes	No	NA	No	c.1750delG (p.Val584Trpfs*37)	
11	58	Male	Yes	No	NA	No	c.1750delG (p.Val584Trpfs*37)	PMID:25683118
12	28	Female	Yes	No	NA	No	c.607dup (p.Ile203Asnfs*8)	
13	3	Female	Yes	No	NA	No	c.424A>T (p.Lys142Ter)	PMID:25683118
14	10	Male	Yes	No	NA	No	c.461dupGCAT (p.Ser154Cysfs*6)	PMID: 28851602
15	5.5	Male	Yes	No	NA	No	c.544G>T (p.Glu182Ter)	
16	Child baring age	Female	Yes	No	NA	No	c.544G>T (p.Glu182Ter)	PMID:31392520
17	30	Male	Yes	No	NA	No	c.507_508ins (p.Glu172Argfs*11)	PMID: 30656735
18	5	Female	Yes	No	NA	No	c.571G>T (p.Gly191Ter)	PMID:32918489
19	5	Female	Yes	No	NA	No	c.565-11A>G	PMID:33010050
20	11	Female	Yes	No	NA	No	c.1423-6G>A (p.Asp475IlefsTer3)	PMID: 33410500

21	9	Male	Yes	No	NA	No	Compound heterozygous *CAST* variants [maternal c.1897-7A>G (p.Leu570) and paternal c.1148T>G (p.Leu383*)] predicted to cause premature termination	PMID:37140444
22	11	Male	Yes	No	NA	No	chr5:g.96748570_96748571insT	PMID:37317743
23	4	Femal e	Yes	No	NA	No	c.571G>T (p.Gly191Ter)	PMID:40387456
24	10	Femal e	Yes	No	NA	No	c.571G>T (p.Gly191Ter)	PMID:40387456
25	2	Male	Yes	No	NA	No	c.706delA (p.Met236TrpfsTer8)	PMID: 39931923
26	6	Femal e	Yes	No	NA	No	c.636dupA (p.Glu213ArgfsTer12)	PMID: 39931923

* The index had no cardiac findings. Seven of his family members who were affected by PLACK syndrome had sudden cardiac death

Methods:

Sanger sequencing

The genetic material from the affected individuals, their parents and siblings, was amplified using PCR with primers. The CAST region was amplified using gene-specific primers (FW: **ATTGTTCGGGCTCCAGAGAT**, RV: **TCGGTTTTGTAATGTGCAGACT** , resulting in an amplicon of approximately **350 base pairs,** *CAST* (NM_001750.7) gene identified by Ensembl Genome Browser (Ensembl URL: https://asia.ensembl.org/Homo_sapiens/Info/Index). PCR was carried out using standard conditions in a final volume of 25 µl with around 10 ng of genetic material. The purified PCR product covering the segment of the chosen variant was sequenced using the dideoxy chain-termination method with an ABI Prism Big Dye Terminator v3.1 Cycle Sequencing Kit and analyzed on an ABI 3730XL capillary sequencer (Applied Biosystems, CA, USA). Sequence analysis was conducted using the SeqMan 6.1 module of the Lasergene (DNA Star Inc. WI, USA) software package and compared to the reference GenBank sequence.

Genome sequencing and variant annotation

Genomic DNA was extracted from the index case and family members as indicated. Illumina DNA PCR-Free Prep kit was used, DNA were sequenced using the NovaSeq 6000 sequencing (Illumina, Inc, USA). The DNA sequence was mapped to and analyzed in comparison with the published human genome build Genome Reference Consortium Human Build 38 (GRCh38) using a local installation of the Illumina DRAGEN pipeline. Quality of sequenced data were assessed for the average depth of coverage and data quality threshold values. Sequence changes in this individual were compared to the other provided family members and population databases. Sequence variants are validated by Sanger sequence analysis (please see above). The DRAGEN Copy Number Variant (CNV) pipeline calls CNV events utilizing next-generation sequencing (NGS) data, a reference-based normalization algorithm that uses additional matched normal samples to establish a baseline level from which to call CNV events. The values below represent metrics from this individual's genome sequencing. Mean Depth of Coverage is > 30X Quality threshold is > 90%. As described above, the internally validated method for NGS and variant annotation, was adopted utilizing the track record of the performance of our CAP-accredited molecular diagnostic facility.
